# The rise of the machines: are robots the future of renal artery aneurysm repair? A systematic review

**DOI:** 10.1007/s13304-024-01971-8

**Published:** 2024-09-16

**Authors:** Paweł Marek Łajczak, Przemysław Nowakowski, Kamil Jóźwik

**Affiliations:** Zbigniew Religa Student Scientific Club, Department of Biophysics, Faculty of Medical Sciences in Zabrze, Medical University of Silesia, Katowice, Poland

**Keywords:** Renal aneurysm, Robot-assisted surgery, Vascular surgery, Da Vinci, Aneurysm

## Abstract

Renal artery aneurysms (RAAs) are a rare vascular condition. Robot-assisted surgery offers a minimally invasive approach for RAA repair, potentially improving surgical outcomes. This review investigates the current evidence on the effectiveness and limitations of this technique. A systematic search following PRISMA guidelines identified relevant studies across five electronic databases. Studies investigating the use of robot-assisted surgery for RAA repair were included. The review identified 11 studies encompassing a total of 23 patients. Procedures included aneurysmectomy, end-to-end anastomosis, prosthetic graft repair, and even coil embolization. All surgeries were successful, with only minor complications reported in four cases. Robot-assisted RAA repair shows promise as a minimally invasive approach with encouraging preliminary outcomes. However, the limited data come from small studies. Future advancements in robotic technology hold the potential to optimize this approach for improved patient care.

## Introduction

Renal artery aneurysm (RAA) is a weakening and bulging of the arterial wall supplying the kidneys. Despite being the second most common visceral aneurysm, RAA is a relatively uncommon condition, affecting approximately 0.1% of the population, primarily women over 50 years old [1, 2].

Most RAAs are asymptomatic. However, hypertension is a frequently observed clinical presentation, affecting roughly 75% of RAA cases [3]. The most severe complication associated with RAAs is aneurysm rupture. Bilateral involvement is observed in approximately 20% of RAAs. Saccular aneurysms are the most prevalent type, and fibromuscular dysplasia is a contributing factor in over one-third of cases [3].

Diagnostic modalities for RAAs include computed tomography (CT) scans and angiography, which aid in assessing the size and morphology of the aneurysm (refer to Rundback classification) [3, 4].

Management of RAAs is individualized and considers factors such as blood pressure control, pregnancy status, aneurysm size and morphology, patient age, and gender [5].

Robotic surgery has emerged as a promising technology in the field of vascular surgery, offering enhanced precision and control during procedures. Introduced in 1985, the Puma 560 system, initially designed for brain biopsy procedures, paved the way for the development of robot-assisted (RA) surgery in other areas. These robotic systems mitigate physiological limitations of surgeons, such as hand tremor and fatigue. Their application is expanding into vascular surgery, including procedures on abdominal and neurological blood vessels, particularly where high precision is required [6].

The Society for Vascular Surgery guidelines published in 2020 suggest considering robotic assistance for RAA repair; however, the supporting evidence remains limited [7]. Since then, no systematic reviews have been published.

This paper aims to comprehensively synthesize current knowledge on the management of RAAs with robotic assistance. We will analyze the effectiveness of this technique, explore the employed methods, discuss its limitations, and examine future directions. This information can contribute to the broader adoption of this technology.

Due to the relative uncommon occurrence of the RAAs and novelty of robotic-assistance surgery in this aneurysm type, cohort studies and randomized trials are notably absent in the literature. Our systematic synthesis will mostly include case reports of single patients and case series, which are for the moment primary source of the information. Nevertheless, this synthesis based on all currently available evidence may provide guidance in future studies and technological advances.

## Methodology

This systematic review adheres to the Preferred Reporting Items for Systematic Reviews and Meta-Analyses (PRISMA) guidelines to ensure transparency and minimize bias in the selection and analysis of studies [8].

### –Research question

The main objective of this paper was to analyze the surgical effectiveness, safety and outcomes of robot-assisted intervention for renal artery aneurysm management: (1) What are the surgical outcomes of RA RAA intervention? (2) What are the patient outcomes during and after RA RAA surgery? (3) What are the current limitations of this technology in RAA interventions? (4) What are the potential advantages and disadvantages compared to traditional (laparoscopic and open) methods?

### –Search strategy

We searched five electronic databases (PubMed, Embase, Web of Science, Scopus, and Cochrane Reviews) for relevant publications. We employed a comprehensive search strategy utilizing medical subject headings (MeSH) terms such as “renal artery repair,” “renal artery aneurysm,” “robot,” “robotic,” and “robot-assisted.” The full search strategy is provided as an appendix to this paper. We deliberately avoided applying filters for publication date, language, or article type to minimize potential bias and maximize the capture of relevant studies. A systematic search included papers published from default database minimum to 13th April 2024.

### –Inclusion and exclusion criteria

We have only included original research articles, which have applied robotic-assistance technology in the renal artery aneurysm interventions. Due to the limited amount of research within this field, only case reports and case series were included. We have excluded non-original articles (abstracts, reviews, editorials, and letters). Articles not published in English were translated using Gemini AI (Google Inc.) to facilitate inclusion. No publication year limit was established, as we expected small number of papers.

### –Study screening

Articles were imported into ZOTERO bibliography manager software. Two independent reviewers screened the retrieved studies. Disagreements were resolved through mutual consensus.

### –Data extraction

Following article selection, two independent reviewers extracted data from the included studies. Extracted data included:

- Country of origin and publication date
- Robotic system utilized
- Patient demographics (number, sex, and age)
- Complications (if any)
- Aneurysm characteristics (size, location, and type)
- Surgical procedures performed
- Intraoperative outcomes (mean blood loss, operative time, warm ischemia time)
- Postoperative outcomes (length of hospital stay)

Any discrepancies in data extraction were resolved through discussion between the two authors.

### –Bias assessment

We have evaluated bias of case reports and series by a tool proposed by Murad et al. [9]. Each study was evaluated in four domains—selection, ascertainment, causality, and reporting. Notably, while this tool provides a total of eight questions, three of them (#4, #5, and #6) were not used, as they were intended for cases of adverse drug events.

### –Synthesis methods

Due to the presence of mostly single-patient case reports, meta-analysis of the included studies would not be appropriate [9]. Therefore, a narrative synthesis of the results was performed with overall summary of the results for given outcomes and characteristics of the studies.

### –GRADE

Due to the retrospective nature of the included studies, absence of direct comparisons, lack of randomization/standardized selection criteria, and small sample sizes, formal GRADE analysis was not deemed appropriate and, therefore, not performed.

### –Software and statistics

A R statistical software (R 4.3.1) was used to perform statistical analyses, which included calculation of mean and standard deviation when these values were not provided in the paper. ZOTERO (version 6.0.36) bibliography manager was used during article screening process.

## Results

**Fig. 1:**
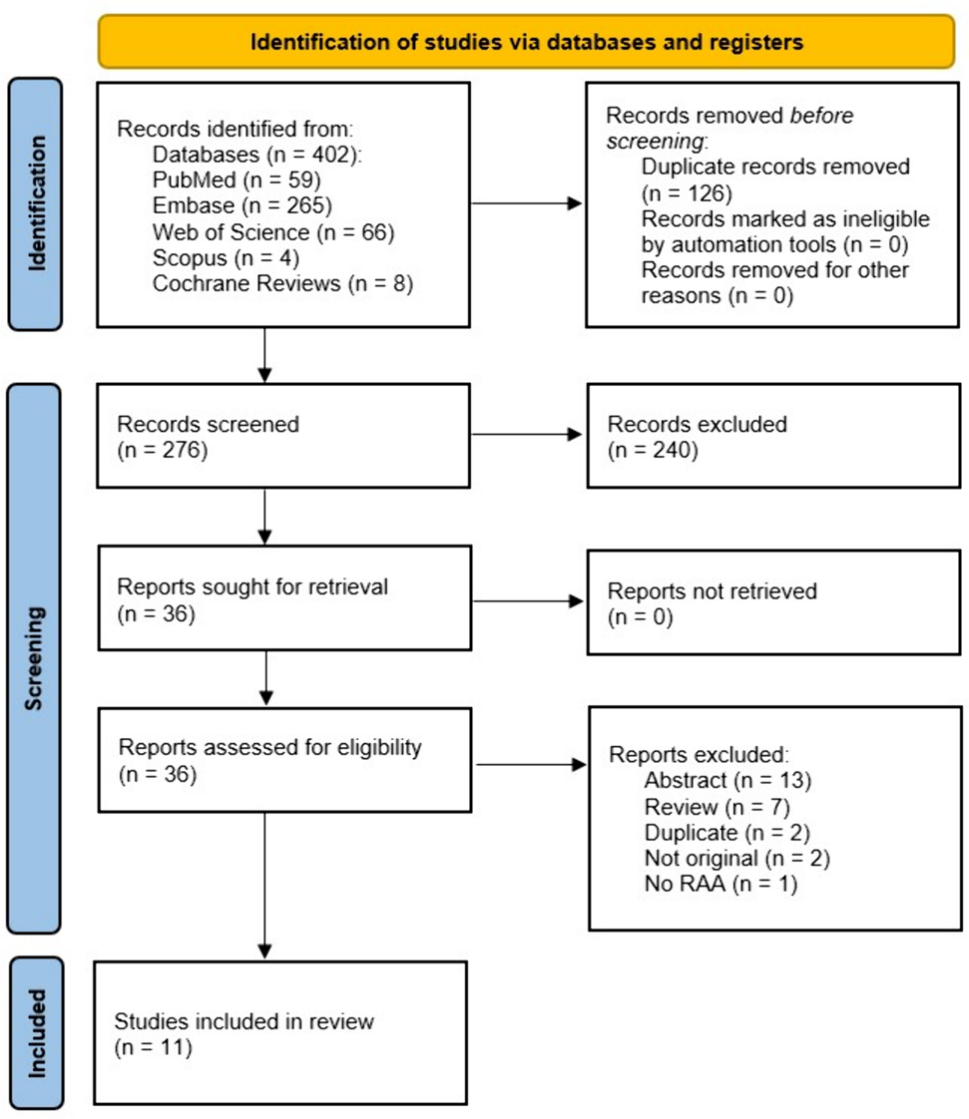
PRISMA flow diagram

A systematic search was conducted on April 13, 2024, across five electronic databases: PubMed, Embase, Web of Science, Scopus, and Cochrane Reviews. The search strategy yielded a total of 402 results (59 from PubMed, 265 from Embase, 66 from Web of Science, 4 from Scopus, and 8 from Cochrane Reviews). Duplicate entries (*n* = 126) identified by Zotero were removed before further analysis. Following title and abstract screening, 240 irrelevant articles were excluded, leaving 36 articles for full-text review. Ultimately, 11 articles met the inclusion criteria and were included in the final analysis (reference list provided) [10–20]. From the 25 excluded papers, there were 13 abstracts, 7 review papers, 2 duplicates, 2 were not original (editorial), and 1 has not included renal aneurysm (Fig. 1).

### –Characteristics of included studies

The 11 included studies originated from various countries, with 5 from the USA, 2 from China, and 1 each from France, Canada, Chile, and Italy (collaboration with USA). Robotic surgical systems were described in all but one study. The da Vinci Surgical System was the most frequently used platform (*n* = 9), with variations including da Vinci Xi (*n* = 2), da Vinci Si (*n* = 2), and da Vinci S-HD (*n* = 1). Abreu et al. have used two surgical systems—da Vinci Xi and da Vinci Si. One study utilized the Magellan Robotic system.

A total of 23 patients underwent robot-assisted RAA procedures in the included studies. The majority (69.6%) were female. Aneurysm characteristics (size, location, and type) and surgical procedures varied across studies. Aneurysmorrhaphy, aneurysmectomy, end-to-end anastomosis, prosthetic graft repair, and coil embolization (one case) were the reported procedures.

### Bias assessment

We have evaluated bias with the tool provided by Murad et al. [9]. We have not found major concerns in ascertainment and reporting sections. Major concerns in the included studies were found in selection domain, as studies often have not reported, if more cases of RA RAA were available in their center. This raises bias, as potentially unfavorable results could be masked by the authors. Additionally, some studies reported relatively short follow-ups, which could overestimate favorable results of RA surgery. Therefore, we have raised concerns in causality domain. No study in this systematic review was assessed as low bias. The results of the bias assessment are presented within Table 1

**Table 1:** Bias assessment

Study	D1	D2	D3	D4	D5	D6	D7	D8	Overall
Grandhomme	No	Yes	Yes	Not evaluated			No	Yes	High
Abreu	No	Yes	Yes				Yes	Yes	Moderate
Gheza	No	Yes	Yes				No	Yes	High
Wu	No	Yes	Yes				No	Yes	High
Giulianotti	No	Yes	Yes				Yes	Yes	Moderate
Samarasekera	No	Yes	Yes				No	Yes	High
Luke	No	Yes	Yes				No	Yes	High
Wei	No	Yes	Yes				No	Yes	High
Castillo	No	Yes	Yes				No	Yes	High
Long	No	Yes	Yes				Yes	Yes	Moderate
Aziz	No	Yes	Yes				Yes	Yes	Moderate

Table Note: Questions from Murad et al. [9]:

1. Does the patient(s) represent(s) the whole experience of the investigator (centre) or is the selection method unclear to the extent that other patients with similar presentation may not have been reported?

2. Was the exposure adequately ascertained?

3. Was the outcome adequately ascertained?

4. Were other alternative causes that may explain the observation ruled out? (Not used).

5. Was there a challenge/rechallenge phenomenon? (Not used).

6. Was there a dose–response effect? (Not used).

7. Was follow-up long enough for outcomes to occur?

8. Is the case(s) described with sufficient details to allow other investigators to replicate the research or to allow practitioners make inferences related to their own practice?

### –Outcomes

Data were extracted and analyzed for relevant surgical outcomes. Mean operative time was 261.4 ± 82.8 min for the 11 patients reported across 7 studies. Mean blood loss was 173.6 ± 174.1 mL for the same 11 patients. Mean warm ischemia time was 39.8 ± 20.4 min. Data on post-operative hospital stay (*n* = 8 patients) showed a mean of 3.25 ± 1.6 days. Time to regular diet was reported for seven patients with a mean of 1.7 ± 0.5 days. Four studies reported pneumoperitoneum pressure, with a mean value of 13.8 ± 1.1 mmHg. Complications were reported in three studies (*n* = 4), including hematocrit drop requiring transfusion, elevated creatinine levels, reconstructed branch stenosis, and mild cellulitis. Abreu et al. have provided median values for outcomes, without providing patient data, hence this study was not included in the calculation of mean values. A comprehensive overview of included studies is presented in Tables 2 and 3.

**Table 2:** Characteristics of included studies

Study	Robot	Patients, Sex (F—Female), Age	Complications	Size	Procedure
Grandhomme et al. [10], France 2021	Da Vinci Xi	1, 1F, 45	None	20 mm, left renal trifurcation (saccular)	In-situ Aneurysmorrhaphy with patch closure; Retrocolic
Abreu et al. [11], USA 2019	Da Vinci Si and Da Vinci Xi	9 (8 underwent repair procedures), 7F, 54 (37–78)—One patient underwent nephrectomy, because repair of RAA was not feasible	Hematocrit drop requiring blood transfusion (× 1)	2.2 (1.8–3) cm; Left (× 5), Right (× 5); Main Bifurcation (× 4), First Branch (× 2), Anterior Segmental Branches (× 2), Main Trifurcation (× 1), Bifurcation of Lower Artery (× 1); Saccular (× 8), Fusiform (× 2); Calcification (× 5); Expanding (× 3)	Aneurysmectomy with repair (× 5), Excision with E-E anastomosis (× 2), Aneurysmectomy with branch reimplantation (× 1), Prosthetic interposition graft repair (× 1); Transperitoneal
Gheza et al. [12], USA 2013	Da Vinci	1, 1F, 41	None	20 mm, right renal artery (saccular)	Y-shaped autologous saphenous graft; Transperitoneal
Wu et al. [13], China 2021	Da Vinci	1, 0F, 58	None	8.6 mm, right distal middle renal artery	Aneurysmectomy with E-E anastomosis; Retroperitoneal
Giulianotti et al. [14], Italy/USA 2010	Da Vinci	5, 5F, 63.8 ± 7.7	Postoperative serum creatinine level elevated (× 1), Stenosis of reconstructed branch (× 1)	19.4 ± 6.2 mm; Left (× 3), Right (× 2); Main bifurcation (× 3), Main trunk (× 1), First posterior branch (× 1)	Aneurysmectomy + Y saphenous vein graft (× 4), Aneurysmectomy with E-E anastomosis; Transperitoneal
Samarasekera et al. [15], USA 2013	Da Vinci Si	1, 0F, 35	None	16 mm right in midpolar region (saccular)	Aneurysmorrhaphy + direct closure; Transperitoneal
Luke et al. [16], Canada 2006	Da Vinci	1, 0F, 54	Mild cellulitis	25 mm, left anterior	Aneurysmectomy + E-E anastomosis; Transperitoneal
Wei et al. [17], China 2017	Da Vinci	1, 0F, 64	None	Multiple (× 2), 18 and 12 mm, right in primary bifurcation (saccular)	Aneurysmorrhaphy + direct closure; Transperitoneal
Castillo et al. [18], Chile 2013	Da Vinci S-HD	1, 0F, 51	None	Multiple (× 3), 42 mm, right middle third (saccular)	Selective ligation
Long et al. [19], USA 2017	Unknown	1, 1F, 38	None	38 × 36 × 32 mm, right	Aneurysmorrhaphy
Aziz et al. [20], USA 2018	Magellan Robotic System	1, 1F, 24	None	25 mm, left hilum (saccular)	Coil Embolization

**Table 3:** Supplementary information

Study	Operation time (min)	Blood loss (mL)	Warm ischemia time (min)	Hospital stay (days)	Regular diet (days)	Pressure pneumo-peritoneum (mm Hg)
Grandhomme	210	100	38	3	N/A	14
Abreu	228 (180–360)	100 (25–400)	26 (19–32)	3 (2–6)	N/A	N/A
Gheza	350	200	82	4	N/A	N/A
Wu	120	50	25	5	2	15
Giulianotti	288 ± 63.1	100 ± 100	32.8 ± 18.7	5.6 (3–7)	1.6 ± 0.49	14
Samarasekera	240	260	44	N/A	N/A	N/A
Luke	360	650	59	3	2	N/A
Wei	155	150	26	6	N/A	12
Castillo	N/A	N/A	N/A	2	N/A	N/A
Long	N/A	N/A	N/A	2	N/A	N/A
Aziz	N/A	N/A	N/A	1	N/A	N/A

### –Analysis of the included studies

Luke et al. reported the first-ever case of RAL RAA in a 54-year-old patient with a left-sided saccular aneurysm unsuitable for endovascular intervention [16]. Due to the challenging location, a saphenous vein graft was harvested endoscopically using the da Vinci Surgical System. The procedure involved occluding the main arterial and venous branches with bulldog clamps, followed by aneurysmectomy and end-to-end anastomosis. While the surgery lasted 6 h, only a minor case of cellulitis occurred, which was treated with antibiotics.

Abreu et al. conducted the largest robotic trial, involving two experienced surgeons with over 2000 robotic procedures [11]. Nine patients with ten RAAs were planned for repair using the da Vinci Xi or Si systems. However, one patient underwent nephrectomy due to unfeasible RAA repair. A six-port transperitoneal approach was used, with various techniques depending on RAA calcification. Tailored excision and repair, excision with end-to-end anastomosis, branch reimplantation, and prosthetic graft reconstruction were employed. Intraoperative assessments included Doppler ultrasound or indocyanine green dye with firefly function to evaluate renal function after intervention. Median operation time was 3.8 h, warm ischemia time was 26 min, and median hospital stay was 3 days. Only one patient experienced a drop in hematocrit requiring a blood transfusion, and no other complications were reported.

Grandhomme et al. presented the first reported case of robotic patch reconstruction with the da Vinci Xi system [10]. A 45-year-old patient with a 20 mm left renal artery aneurysm unsuitable for endovascular embolization (due to its location at the trifurcation) underwent RAL RAA. With the patient positioned on their left side, a left retrocolic approach was chosen. Aneurysmorrhaphy was performed, followed by closure using a Dacron patch. Warm ischemia time was 38 min, total operation time was 210 min, and blood loss was minimal (100 mL). There were no complications during or after surgery, and the patient was discharged on the third day.

Wu et al. described the first successful application of the retroperitoneal approach for RAL RAA, citing its advantage of direct access to the renal artery [13]. A 58-year-old patient with an 8.6 mm aneurysm underwent this procedure. An inflated balloon created a retroperitoneal space, followed by opening the Gerota fascia. Aneurysmectomy with end-to-end anastomosis was performed without cold renal perfusion due to the short ischemia time (25 min). No complications were reported post-surgery, nor during the 2.5-month follow-up.

Wei et al. reported the treatment of a 64-year-old patient with two saccular aneurysms (18 mm and 12 mm) at the primary bifurcation of the right renal artery [17]. RAL aneurysmorrhaphy was performed using the da Vinci Surgical System with no postoperative complications.

In contrast to the previous RAL RAA cases, Aziz et al. described the first-ever application of an endovascular robotic system for a patient unsuitable for open surgery [20]. Due to the challenging location of the left renal artery hilum aneurysm in a 24-year-old patient, coil embolization was performed with the Magellan Robotic System. This system features a robotic arm controlled from a shielded room to minimize radiation exposure. The robotic arm's maneuverability allows for precise navigation of instruments within the vessels. The procedure involved accessing the right common femoral artery, maneuvering a guidewire, catheter, and sheath into the puncture site, and positioning them at the aneurysm neck for embolization with EV3 Concierto microcoils. Following successful embolization, the patient was discharged the next day without complications, and follow-up confirmed successful aneurysm exclusion with normal kidney function.

Included studies demonstrated that the effectiveness of robotically-assisted renal aneurysm interventions is generally successful, with no conversions to manual surgeries or major complications. Various surgical techniques (aneurysmectomy, end-to-end anastomosis, and coil embolization) were successfully accomplished, showing potential in RAA management. However, it should be emphasized, that RA management is not fully free of complications, as minor events (hematocrit drop, elevated creatine level, stenosis or cellulitis) occurred among the studies.

Patient outcomes were favorable among included case reports. Mean surgery time was 261.4 ± 82.8 min, and mean blood loss was 173.6 ± 174.1 mL. Postoperative recovery time was also reported positively, with a mean hospital stay of 3.25 ± 1.6 days and a mean time to regular diet of 1.7 ± 0.5 days. These results support clinical acceptance of robotic devices, as perioperative and postoperative outcomes remain acceptable.

While papers included in this systematic review have not directly evaluated surgical robots to open and manual techniques, there are several advantages over conventional methods. The use of robotic systems allows for millimeter precision, which is unachievable by surgeons due to the physiological factors (limited eye resolution and hand tremor). This results in lesser complications caused by damage to the surrounding structures during the surgery, smaller blood loss, and shorter duration of hospital stay.

However, robotic surgery requires time-consuming specialized training. While there was no cost-effectiveness analysis, the high purchase costs may result in economical ineffectiveness in small surgical centers, and adoption in such healthcare facilities might be limited. The comparisons should be evaluated in detail in future trials, as current evidence relies on case reports.

## Discussion

Renal artery aneurysm, while being relatively uncommon, may lead to life-threatening consequences in case of the rupture. Proper early diagnosis and treatment are crucial for the minimization of the rupture risk. Currently ongoing debate of the application of robot assisted surgery, discusses its effectiveness in clinical practice. In our review, through the PRISMA guidelines, we have systematically searched major medical databases, in order to find studies applying this technology into research studies.

### –Benefits of RA

Robots offer various benefits. First of all, elimination of the physiological factors, which include surgeon’s hand tremor, fatigue, and lower eye accuracy, allow for more precise movements, and improved control of the surgical instruments during the RAA interventions. The more precise the surgery is, the lesser is the risk of intra-/and post-operative complications. The elimination of additional adverse events results in the lower hospital stay, and minimizes the number of additional interventions, benefiting both patients and healthcare providers, as it decreases the logistical and economic costs of the provided services. Additionally, the minimally invasive approach offers greater cosmetic result after the surgery, reducing psychological concerns related to the visual result [21].

Various meta-analyses reported that RA surgery, compared to the traditional surgery, allows for lower blood loss during the surgery, decreased number of the complications, as well as better cosmetic outcomes, which are especially important for the patient [22–24]. These benefits should be analyzed in the future pair-wise trials of the RAA management.

RA surgery offers more advanced visualization of the surgical table. Advanced 3D imaging allows to perform more advanced maneuvers inside the body. Additionally, flexibility, precision, and huge control of the robotic arm provides an opportunity to conduct complex procedures, which would not be possible for conventional laparoscopic or open surgeries, including difficult location, torturous anatomy, or multiple vessels involved.

### –Limitations of the evidence

However, with these benefits, RA management of RAA is not without any limitations. First of all, we have included only 11 articles in this systematic synthesis, which covered only 23 patients. Therefore, results of this paper should be proceeded with caution. Most of the included papers were case reports of single patients, while none of the studies had more than ten patients. As we have shown in the results section, these studies were prone to bias. The cohort sizes of the included studies highly limits full analysis of this technology. Furthermore, the evidence is limited due to the lack of any pairwise studies, where RA RAA management could be compared to other types of conventional techniques, such as manual laparoscopy, or open surgery. While, as previously mentioned, some studies have shown that robot surgery can be the most beneficial option, here no comparison was performed. Hence, the definitive assessment of benefits over traditional technique remains a question. The overall evidence in this synthesis remains very limited.

Additionally, heterogeneity among the results and methodology was present. Because of inclusion of very low numbers of patients, studies varied in used robot systems (mostly da Vinci), operation, warm ischemia time, blood loss during surgery, approach and procedures during the surgery, limiting the possibility of drawing definitive conclusions.

On the other hand, various approaches including aneurysmectomy, E-E anastomosis, prosthetic graft, or endovascular intervention showed technical success. All performed surgeries were successful, and no major complications or conversions to manual technique were present. This shows the flexibility of robot devices in various types of RAA management. Minor complications, which were reported in four cases, included mild cellulitis, elevated creatinine level, or hematocrit drop suggest the need for future development and improvements of these devices. Mean blood loss during operation, operation time, and hospital stay reported from studies suggest favorable outcome for this technique.

To conduct a full cost-effective analysis, long-term data are required, which is notably lacking at the moment. These parameters could report the durability of RA repairs (i.e. presence of stenosis, or other complications), renal function, and additional interventions. These reports could provide long-term costs of such management, enhancing the cost analysis.

### –Disadvantages of RA surgery

Major limitation is the entry level for such device. While the robotic system may be cost-effective, the purchasing cost may be discouraging [25, 26]. For example, da Vinci surgical system can cost hundreds of thousands of dollars [27]. Another question is if RAA surgeries need specific conditions, to be proven to be cost-effective for the healthcare facility.

Finally, the robots require specialized training. A survey among the Brazilian College of Surgeons analyzed the training on Da Vinci SI robotic system [28]. The average time for pre-clinical training was 116 days. This training requires additional time from surgeon, and expenses on proper education to ensure effectiveness of this method.

### –Future directions

There are many future directions for RA RAA management, however, the most important for now is conducting large-scale pairwise studies. Prospective studies, with detailed selection criteria, randomization, and low risk of bias could fairly report the effectiveness, safety, and comparison to the other methods.

These studies should also include aspects of the cost-effectiveness analysis, such as long-term follow-ups, comparison to manual methods, or number of additional performed procedures. Inclusion of all cost-related aspects of RA RAA management could lead to meaningful cost-effectiveness meta-analysis.

In case of robots itself, the next steps should include single port laparoscopic surgeries, which could even more reduce the invasiveness of the intervention [29].

Another aspect to consider is the reduction of time for warm ischemia. Literature indicates that clamping time is still subject under debate, and suggest between 30 and 60 min, while some studies had nearly double times compared to others (Table 3) [30–32]. The pressure of WIT, especially in the case of multiple aneurysms in the single patients, creates problems with dealing with complex anatomies during the suturing and angioplasty. Surgical robots, with more advanced 3D optical imaging systems and flexible wristed instruments could reduce this time.

Finally, more endovascular techniques should be performed in the future trials and robot developments. Currently, because of the difficult locations of the RAA, RAL approach is more commonly chosen as seen in this synthesis, especially in the aneurysms located close to the hilum. RA endovascular approach could reduce post-operative pain, lower risk of complications, minimize the radiation exposure to the staff, while at the same time, be capable of dealing with difficult locations of RAA.

### –Limitations of the review process

This systematic review is mostly based on single-patient case reports and case series. Due to the heterogeneity of the results, and small number of patients, a quantitative analysis was not performed in this systematic review. Additionally, we have applied narrative synthesis of the results. GRADE evidence assessment was not performed due to the reasons mentioned above. Our systematic review has not included databases from China, which could possess potential studies related to RA RAA management. However, as we do not possess access to such websites, the search was not performed.

## Conclusions

Robot-assisted RAA repair shows promise as a minimally invasive approach with encouraging preliminary outcomes. However, the limited data come from small, non-randomized studies with significant methodological variations. Additionally, the high cost of robotic systems and surgeon training pose challenges. Larger comparative studies and cost-effectiveness analyses are crucial to confirm its effectiveness and wider adoption. Future advancements in robotic technology hold the potential to optimize this approach for improved patient care.

## Data Availability

All analyzed and extracted data were presented within the manuscript.

## Appendix

PubMed–59—(Renal aneurysm repair OR Renal aneurysm OR renal artery aneurysm OR Renal aneurysmectomy) AND (robot OR robotic OR robot-assisted OR robot assisted).

Embase–265—(Renal aneurysm repair OR Renal aneurysm OR renal artery aneurysm OR Renal aneurysmectomy) AND (robot OR robotic OR robot-assisted OR robot assisted).

Web of Science–66—(Renal aneurysm repair OR Renal aneurysm OR renal artery aneurysm OR Renal aneurysmectomy) AND (robot OR robotic OR robot-assisted OR robot assisted).

Scopus–4—(Renal aneurysm repair OR Renal aneurysm OR renal artery aneurysm OR Renal aneurysmectomy) AND (robot OR robotic OR robot-assisted OR robot assisted).

Cochrane Reviews–8—(Renal aneurysm repair OR Renal aneurysm OR renal artery aneurysm OR Renal aneurysmectomy) AND (robot OR robotic OR robot-assisted OR robot assisted).
